# Phase I study evaluating the treatment of patients with locally advanced pancreatic cancer with carbon ion radiotherapy: the PHOENIX-01 trial

**DOI:** 10.1186/1471-2407-13-419

**Published:** 2013-09-14

**Authors:** Stephanie E Combs, Daniel Habermehl, Meinhard Kieser, Constantin Dreher, Jens Werner, Renate Haselmann, Oliver Jäkel, Dirk Jäger, Markus W Büchler, Jürgen Debus

**Affiliations:** 1 Department of Radiation Oncology, University Hospital of Heidelberg, Im Neuenheimer Feld 400, 69120 Heidelberg, Germany; 2 Institute of Medical Biometry and Informatics, University of Heidelberg, Im Neuenheimer Feld 305, 69120 Heidelberg, Germany; 3 Department of Visceral Surgery, University Hospital of Heidelberg, Im Neuenheimer Feld 110, 69120 Heidelberg, Germany; 4 Department of Oncology, National Center for Tumor Diseases (NCT), Im Neuenheimer Feld 470, 69120 Heidelberg, Germany; 5 Heidelberger Ionenstrahl Therapiezentrum (HIT), Im Neuenheimer Feld 450, 69120 Heidelberg, Germany; 6 Nationales Centrum für Tumorerkrankungen (NCT), Medizinische Onkologie, Im Neuenheimer Feld 360, 69120 Heidelberg, Germany

## Abstract

**Background:**

Treatment options for patients with locally advanced pancreatic cancer include surgery, chemotherapy as well as radiotherapy. In many cases, surgical resection is not possible, and therefore treatment alternatives have to be performed. Chemoradiation has been established as a convincing treatment alternative for locally advanced pancreatic cancer. Carbon ions offer physical and biological characteristics. Due to their inverted dose profile and the high local dose deposition within the Bragg peak precise dose application and sparing of normal tissue is possible. Moreover, in comparison to photons, carbon ions offer an increased relative biological effectiveness (RBE), which can be calculated between 1.16 and 2.46 depending on the pancreatic cancer cell line as well as the endpoint analyzed. Japanese Data on the evaluation of carbon ion radiation therapy showed promising results for patients with pancreatic cancer.

**Methods and design:**

The present PHOENIX-01 trial evaluates carbon ion radiotherapy using the active rasterscanning technique in patients with advanced pancreatic cancer in combination with weekly gemcitabine and adjuvant gemcitabine. Primary endpoint is toxicity, secondary endpoints are overall survival, progression-free survival and response.

**Discussion:**

The physical and biological properties of the carbon ion beam promise to improve the therapeutic ratio in patients with pancreatic cancer: Due to the inverted dose profile dose deposition in the entry channel of the beam leads to sparing of normal tissue; the Bragg peak can be directed into the defined target volume, and the sharp dose fall-off thereafter again spares normal tissue behind the target volume. The higher RBE of carbon ions, which has been shown also for pancreatic cancer cell lines in the preclinical setting, is likely to contribute to an increase in local control, and perhaps in OS. Early data from Japanese centers have shown promising results. In conclusion, this is the first trial to evaluate actively delivered carbon ion beams in patients with locally advanced pancreatic cancer within a dose-escalation strategy.

**Trial registration:**

NCT01795274

## Background

Cancer of the pancreas is the fourth most common cause of cancer-related death in men and women. Ductal adenocarcinoma is the most common variant in over 90% of all pancreatic malignancies. Patients commonly present with syptoms such as weight loss, jaundice, pain, dyspepsia or stool irregularities.

Surgical resection is considered the treatment of choice; in over 80% of all patients surgery alone does not convert into long-term tumor control. In highly specialized centers, surgery associated mortality has been reduced to < 5%, but even in these centers overall survival after surgery alone remains to be around 20 months, with a five year survival rate of about 20% [1,2]. Several studies have shown that strong prognostic factors are resection status, presence of lymph-node metastases, tumor size as well as tumor DNA content [3-6]; with respect to resection status, only R0-resection seems for provide a major benefit as compared to R1 or R2 resections.

Pancreatic cancer is commonly classified as resectable, borderline resectable or unresectable. Interdisciplinary discussion on a case-by-case basis is recommended for all patients with respect to optimal treatment of patients; individual decisions for local treatment may vary depending on tumor size, symptoms or other.

Special focus has been set on the group of patients with non-metastasized, but locally advanced and thus non-resectable or borderline resectable patients. In these cases, alternatives to radical surgery must be evaluated. In the past, chemotherapy alone was administered, usually with gemcitabine or 5-FU.

Several concepts of chemoradiation have been established, and clincial data has shown beneficial results with acceptable toxicity. A GITSG study initially defined the role for radiation and chemotherapy with bolus 5-FU, comparing split-course radiotherapy with 40 Gy with chemotherapy to radiation with 60 Gy in combination with chemotherapy or radiation alone: Median survival could be increased from 22.9 weeks to 42.2 weeks [7]. Subsequent studies aimed at the optimization of 5FU application, and most contemporary studies have left the regimen of split-course radiotherapy. Gemcitabine has also been used as a radiation sensitizer in pancreatic cancer [8-12], and several studies have shown that gemcitabine and radiation seem to be equieffective to 5-FU and radiation [10,13].

To date, no specific randomized trials evaluating the effect of chemoradiation have been performed. Results from a prospective study evaluated clinical outcome after radiation, gemcitabine and cisplatinum in patients with locally advanced pancreatic adenocarcinoma. The regimen showed acceptable toxicity, but no benefit compared to other single-agent chemotherapeutic treatments was shown [14]. A retrospective analysis performed by the MD Anderson Cancer Center revealed that in resectable patients preoperative radiation is not disadvantageous, and since postoperative recovery often extends the time until (about 25% of all patients) adjuvant treatment can be initiated [15]. Several other arguments can be brought up supporting radiochemotherapy before any surgical resection: Likelihood of R0 resections due to tumor shrinkage, selection of patients with more stable disease for surgery, treatment of micrometastases at an earlier stage and treatment of tissue not modified by surgery and potentially more sensitive to chemotherapy and radiation [9,16,17]. The MD Anderson group showed that median survival was 21 months, 31% of all patients demonstrated no evidence of disease, in 132 patients treated with preoperative chemoradiation and subsequent pancreaticoduodenectomy [15]. In our institution we have shown that 26% of patients with locally advanced pancreatic cancer can undergo secondary resection after gemcitabine-based chemoradiation, and that patients with a complete resection are associated with a relatively good long-term prognosis [18].

The idea to bring locally advanced tumors into a resectable status has been addressed explicitly in several studies [17,19-24]. There is evidence that the rate of margin-free resection can be increased by preoperative chemoradiation [25]; however, no randomized trials have confirmed this hypothesis. Palmer and colleagues could show that gemcitabine-based chemotherapy given as neoadjuvant therapy for potentially resectable pancreatic cancer lead to an increase in resectability as compared to gemcitabine alone [21]. On the other hand, some authors have shown that even after combined radiation and chemotherapy the rate of potentially resectable patients did not increase, and a substantial amount of patients presented with advanced disease 4–6 weeks after completion of radiochemotherapy, when restaging was performed [8,26]. At the Heidelberg Center, a novel approach of using modern intensity modulated radiotherapy (IMRT) in combination with gemcitabine and the EGFR-antibody cetuximab has been performed with a comparable intent [27]. The final study results are will be reported soon, the study has finished recruitment.

To date, within treatment recommendations for locally advanced pancreatic cancer without distant metastases, radiation or radiation alone can be found as possible approaches. Besides modifying systemic treatment and keeping photon radiation as a constant factor, new radiation modalities such as particle therapy offer distinct physical and biological characteristics and are a promising treatment alternative for patients with pancreatic cancer potentially increasing local responses, leading to higher local tumor control rates, higher rates of resectability, as well as an increase in survival.

Proton as well as carbon ion radiotherapy are characterized by an inverted depth-dose-curve, with low dose deposition within the entry channel, and a defined high local dose deposition in the so called Bragg Peak. Therefore, sparing of normal tissue in front of and behind the defined target volume is possible. More conformal dose delivery with increased sparing of healthy surrounding normal tissue is possible, enabling the application of higher local doses to the target volumes. Carbon ions additionally offer a higher biological effectiveness due to the characteristic and severe radiation damages produced in target tissues. For pancreatic cancer cell lines, RBE for carbon ion beams values between 1.16 and 2.46 have been reported depending on cell line and endpoint [28]. Is has been shown that carbon ion radiotherapy leads to an increase in local control especially in radiation resistant tumors, such as chordomas and chondrosarcomas, adenoidcystic carcinomas, melanomas as well as HCC, as well as pancreatic cancer [29].

At Japanese institutions clinical trials have been conducted to evaluate carbon ion radiotherapy for pancreatic cancer. Between 2000 and 2003, 22 patients with localized, resectable adenocarcinoma of the pancreas were treated with preoperative carbon-ion radiotherapy. Doses between 44.8 Gy E and 48 Gy E in single doses of 2.8 Gy E and 3.0 Gy E were applied. Local control rate was 100%, and overall survival was 59% at 1 year. A subgroup of patients did not receive post-radiotherapeutic resection, which showed a significantly lower outcome (1 year overall survival of 3%) as compared to patients receiving surgery (86% overall survival at 1 year [30]). This was followed by a more hypofractionated regimen in a study with comparable inclusion criteria, increasing applied dose from 30 Gy E to 35.2 Gy E in 8 fractions. Still, no local tumor recurrences were observed, and overall survival at 1 and 5 years in patients treated with surgical resection after preoperative carbon ion radiotherapy was 89% and 51% [30]. Patients with locally advanced and in the first step inoperable pancreatic cancer were included into a Phase I/II trial. Inclusion criteria included patients with histologically confirmed ductal carcinoma of the pancreas, volume of 14 cm or less in diameter, inoperable lesions; patients were treated with increasing doses from 38.4 Gy E to 52.8 Gy E in 12 fractions. Overall survival at 1 year was 60% with a local control rate of 81%, and patients receiving higher doses showed a significant benefit in local control and overall survival [30]. In analogy to treatment schedules with photons, also the combination of chemotherapy with gemcitabine and carbon ions was transferred into a trial protocol: In the first part, a carbon ion dose of 43.2 Gy E in 12 fractions was set as constant, weekly gemcitabine was increased from 400 mg/m^2^ to 1000 m^2^. Acute hematological toxicity as well as non-hematological side effects were low, no grade 4 and 5 toxicities were observed. Only in the 700 mg/m^2^ and 1000 mg/m^2^ gemcitabine arm, 3/6 (50%) and 8/12 (75%) developed grade III hematological toxicity. In all three treatment arms local control was comparable during follow up, but survival was higher with increasing doses of chemotherapy. At present a constant dose of gemcitabine at 1000 mg/m^2^ is applied with a carbon ion dose escalated to 50.4 Gy E [30].

Therefore, in the present PHOENIX-01 trial, carbon ion radiotherapy using the active rasterscanning technique will be evaluated in patients with advanced pancreatic cancer in combination with weekly gemcitabine and adjuvant gemcitabine.

## Methods and design

The purpose of the trial is to evaluate carbon ion radiotherapy in patients with locally advanced pancreatic cancer. With respect to toxicity, the optimal dose of carbon ion radiotherapy will be determined. Therefore, the primary endpoint is toxicity, secondary endpoints are evaluation of progression-free survival, response, and overall survival after carbon ion radiotherapy.

Focus of the analysis is to evaluate safety and efficacy of carbon ion radiotherapy in patients with locally advanced pancreatic cancer. Therefore, the aim of the trial is to observe low rates of toxicity with high local doses due to effect of the altered biology of carbon ions on pancreatic cancer cells as well as the superior physical characteristics.

The primary objective is toxicity of carbon ion radiotherapy. As secondary objectives, imaging response, progression-free survival and overall survival were defined. The trial will be performed as a single-center single-armed Phase I study. Informed consent will be obtained by all patients.

### Treatment schedule

Patients fulfilling the inclusion criteria will be included into the following Phase I dose escalation treatment scheme:

Step 1: 14 × 3 Gy E 42 Gy E

Step 2: 15 × 3 Gy E 45 Gy E

Step 3: 16 × 3 Gy E 49 Gy E

Step 4: 17 × 3 Gy E 51 Gy E

Step 5: 18 × 3 Gy E 54 Gy E

**Figure 1:**
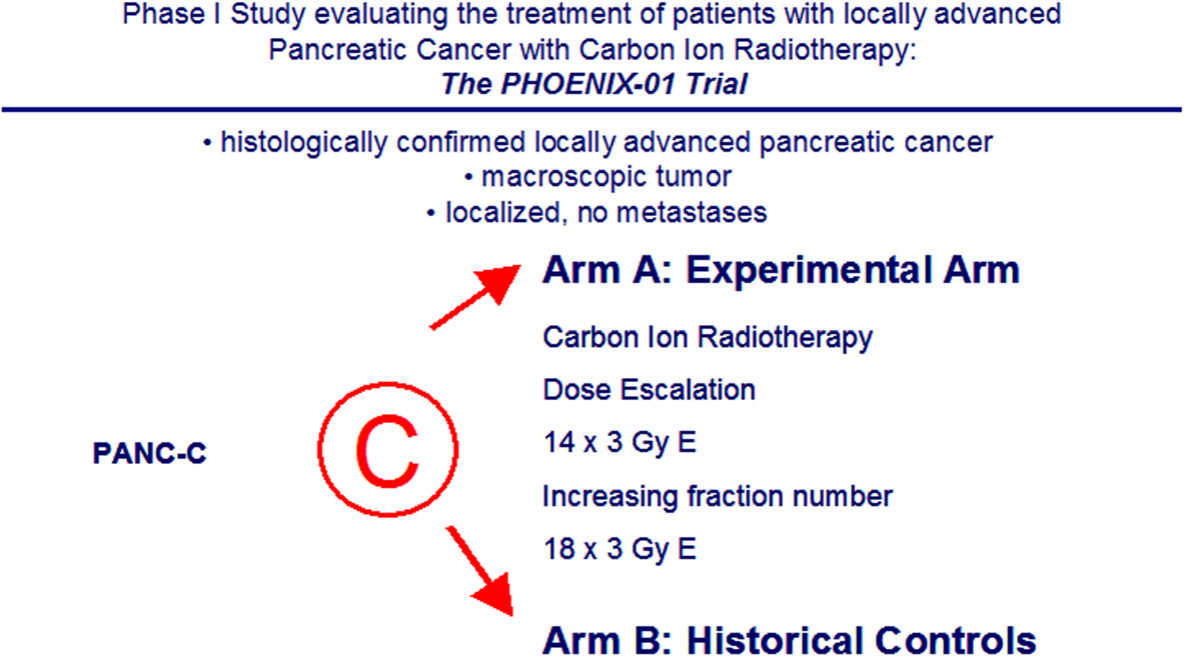
PHOENIX-01-Trial scheme.

Standard chemotherapy with gemcitabine (GEM) 300 mg/m^2^ will be continued during the radiation treatment. The trial scheme is shown in Figure 1.

### Study endpoints and trial duration

The primary endpoint is acute toxicity of carbon ion radiotherapy observed within 3 months of study treatment. Patients are scheduled for follow-up visits every 4 weeks after completion of carbon ion radiotherapy for the first 3 months, thereafter in 2-months intervals or as needed clinically including contrast-enhanced CT or MRI as well as thorough clinical-neurological and haematological assessment. PET imaging can be scheduled to additionally assess tumor response. These follow-up visits are in line with standard care outside of clinical trials. The last patient included into the study will be followed for at least 3 months after study treatment. This is considered the final study visit.

### Patient selection

A maximum of 33 patients should be enrolled in the clinical trial.

Patients with the diagnosis of locally advanced pancreatic cancer will be evaluated and screened for the protocol. All patients fulfilling the inclusion and exclusion criteria will be informed about the study.

### Inclusion criteria

Patients meeting all of the following criteria will be considered for admission to the trial:

histologically confirmed locally advanced pancreatic cancer or imaging defined pancreatic cancer combined with elevated CA-19-9

macroscopic tumor after biopsy

age ≥ 18 years of age

Karnofsky Performance Score ≥60

for women with childbearing potential, (and men) adequate contraception (sexual abstinence, estrogen- or gestagen containing contraceptive medication etc.)

female participants: No pregnancy present (pregnancy test required)

ability of subject to understand character and individual consequences of the clinical trial

written informed consent (must be available before enrolment in the trial)

### Exclusion criteria

Patients presenting with any of the following criteria will not be included in the trial:

distant metastases

refusal of the patients to take part in the study

previous radiotherapy of the abdomen

patients who have not yet recovered from acute toxicities of prior therapies

known carcinoma < 5 years ago (excluding Carcinoma *in situ* of the cervix, basal cell carcinoma, squamous cell carcinoma of the skin) requiring immediate treatment interfering with study therapy

pregnant or lactating women

participation in another clinical study or observation period of competing trials, respectively

### Radiation therapy: treatment planning & dose prescription

For particle therapy, patients will be immobilized using an individually manufactured body fixation or positioning device including abdominal pressure plates as described in detail previously [31]. For treatment planning, contrast-enhanced CT (3 mm slice thickness) as well as MR-imaging will be performed for optimal target definition. 4D-CT-imaging is considered standard of care for target definition when treating patients with moving organs. Additionally, PET examinations may be included into target volume definition for treatment planning.

Organs at risk such as the intestines, stomach, lungs, kidney, spleen and spinal cord will be contoured. Dose constraints of normal tissue will be respected according to Emami et al. [32].

The Gross Tumor Volume (GTV) will be defined as the area of solid macroscopic tumor contrast enhancement on CT and MR-imaging and/or PET positive areas.

The Clinical Target Volume (CTV) will be defined as the GTV plus a margin of at least 5 mm, however, including the regional lymph nodes and any areas of microscopic spread.

The Planning Target Volumes (PTVs) will include the CTV plus a safety margin accounting for organ motion and setup inaccuracies. This margin will depend on the immobilization system as well as the technical features for motion compensation, i.e. gating, rescanning etc.

Proton and Carbon ion RT planning is performed using the treatment planning software PT-Planning (Siemens, Erlangen, Germany) including biologic plan optimization. Biologically effective dose distributions will be calculated using the a/ß ratio for pancreatic cancer as well as for the endpoint late toxicity.

No interruptions > 4 days between during study treatment are allowed.

Patient positioning prior to particle therapy will be evaluated by comparison of x-rays to the DRRs. Set up deviations >3 mm are corrected prior to radiotherapy.

The intensity-controlled rasterscanning system will be used for beam application. Treatment planning aims in the coverage of the PTV by the 90%-isodose line. Dose specification is based on biologic equivalent dose because of the high relative biologic effectiveness (RBE) of carbon ions, which differs throughout the target volume due to its dependence on various factors. RBE will be calculated at each voxel throughout the target volumes and biological optimization will be performed. The dose prescription used is related to the isoeffective dose Gy E (Cobalt Gray equivalent) using daily fractions of 2 Gy and a weekly fractionation of 5 × 2 Gy.

### Follow-up

Regular follow-up visits will be scheduled every 4 weeks after completion of study treatment for the first 3 months, thereafter in 2-months intervals or as required clinically.

After completion of study treatment, treatment of patients with gemcitabine is recommended. Any systemic treatment or chemotherapy is not part of the clinical trial. Evaluation of surgical resection will be performed 4 weeks after completion of radio-chemotherapy in an interdisciplinary setting.

For tumor progression, treatment alternatives will be evaluated and discussed interdisciplinary considering options of resection, systemic treatment such as chemotherapy, a second course of radiation therapy, or other.

During follow-up, the following parameters for assessment of efficacy will be evaluated:

#### Progression-free survival (PFS) and treatment response

Efficacy of the treatment will be recorded according to the RECIST-Criteria (Revised Guidelines, Version 1.1,2009 [33]):

##### Complete response (CR)

Disappearance of all target lesions.

##### Partial response (PR)

At least 30% decrease in the sum of diameters of target lesions, taking a reference the baseline sum diameters.

##### Progressive disease (PD)

At least a 20% increase in the sum of diameters of target lesions, taking as reference the smallest sum on study (this includes the baseline sum if that is the smallest on study). In addition to the relative increase of 20%, the sum must also demonstrate an absolute increase of at least 5 mm (Note: The appearance of one or more new lesions is also considered progression).

##### Stable disease (SD)

Neither sufficient shrinkage to qualify for PR nor sufficient increase to qualify for PD, taking as reference the smallest sum diameters while on study.

The principal investigator or study co-ordinator may be contacted for further discussion on a case by case basis.

#### Overall survival (OS)

##### Overall survival

Is a secondary endpoint of the study. All patients will be followed until death. The duration of survival is the time interval between initial diagnosis (date of the pathology report) and the date of death due to any cause. Patients not reported dead or lost to follow-up will be censored at the date of the last follow-up examination.

#### Safety parameters

This study will use the International Common Terminology Criteria for Adverse Events (CTCAE) version 4.1 for toxicity and adverse event reporting. A copy or the CTCAE can be accessed from the CTEP home page http://ctep.cancer.gov/protocolDevelopment/electronic_applications/ctc.htm).

Safety and toxicity of the study treatment will be evaluated by clinical examination, haematological evaluation as well as imaging studies (MRI or CT).

Other parameters:

The following parameters will be collected and taken into account in the analyses: age, Karnofsky Performance Score, tumor extent, response to radiation therapy, CEA, CA 19–9 and CA 125.

### Study hypothesis & sample size calculation

It is the aim of this Phase I study to determine the MTD for carbon ion radiotherapy for the treatment of advanced pancreatic cancer. The primary endpoint is the occurrence of a dose limiting toxicity defined as any Grade IV toxicity according to CTCAE Version 4.1, possibly, probably or definitely associated to study treatment and occurring within 3 months after completion of the study treatment.

The calculation of the sample size for the PHOENIX-01 trial is based on the traditional 3 + 3 dose escalation scheme [34] which is conducted as follows:

Patients are treated in cohorts of three each receiving the same dose. For the assessment of a dose limiting toxicity (see definition above) patients are observed for 3 months after application of the study treatment. If none of the three patients of a cohort exhibits a dose limiting toxicity, the next cohort of three patients receives the next higher dose. Otherwise, if at least one patient of a cohort exhibits a dose limiting toxicity, a further cohort of three patients is treated at the same dose level without escalating the dose. If exactly one out of the six patients treated at this dose exhibits a dose limiting toxicity, the trial continues as planned at the next higher dose level. If two or more patients out of the six patients treated at this dose exhibit a dose limiting toxicity, the dose escalation stops at that level and the next lower dose is considered as the MTD. When the escalation has stopped, additional patients will be treated at the MTD until a total of nine patients is reached.

The trial is conducted to determine the MTD of carbon ion radiotherapy by consideration of a total of five dose levels. Therefore, the maximum sample size is 33 patients (four dose levels with a maximum of 6 patients each and 9 patients at the MTD).

Primary endpoint to determine the MTD out of the five investigated dose levels is any Grade IV toxicity according to CTCAE Version 4.1, possibly, probably or definitely associated to study treatment and occurring within 3 months after completion of the study treatment. Secondary endpoints are other safety data on the applied dose levels as well as treatment response, progression-free survival, and overall survival.

### Analysis population

The analysis set for the primary endpoint consists of all patients treated at least once with the study treatment. A maximum total of five analysis sets (one per dose level) will be considered. Likewise, all patients treated at least once with the study treatment will be included in the analysis of the secondary endpoints. Additionally, a maximum total of five analysis sets (one per dose level) will be considered to determine any differences in secondary endpoints between the dose levels applied in this study.

### Statistical methods

#### Confirmatory analysis

No confirmatory statistical analysis will be performed. The MTD is determined according to the traditional 3 + 3 dose escalation scheme for Phase I trials in oncology as described above.

#### Descriptive analysis

Descriptive summary tables for the pooled set of patients as well as separated by dose level will be presented for the baseline patient characteristics as well as for all safety parameters. Absolute and relative frequencies are reported for all toxicities of the CTC list (NCI CTCAE Version 4.1) by distinguishing the grading and the assessment of the relation to treatment. A description of the individual load of toxicity of each patient will be given using individual tabulations which can be supported by graphical methods where required.

Secondary endpoints will be evaluated by calculating appropriate measures of the empirical distribution. Furthermore, methods for time-to-event data will be applied and graphical displays of Kaplan-Meier curves will be shown.

Besides the planned analyses that are performed within the dose escalation scheme to decide about the dosage of the next cohort, no further interim analyses are planned within the PHOENIX-01 study.

## Discussion

About one third of all patients with the primary diagnosis of pancreatic cancer present with non-metastasized, locally advanced disease. Primary curative resection is rarely possible in these patients, however, it is known that complete resection of the tumor is the strongest prognostic factor. Thus, strategies for downsizing of locally advanced tumors have moved into focus: With radiochemotherapy using photons, about 20-30% of all patients will become resectable [2,18,35].

The physical and biological properties of the carbon ion beam promise to improve the therapeutic ratio in patients with pancreatic cancer: Due to the inverted dose profile dose deposition in the entry channel of the beam leads to sparing of normal tissue; the Bragg peak can be directed into the defined target volume, and the sharp dose fall-off thereafter again spares normal tissue behind the target volume. The higher RBE of carbon ions, which has been shown also for pancreatic cancer cell lines in the preclinical setting, is likely to contribute to an increase in local control, and perhaps in OS [28,30,36,37]. Early data from Japanese centers have shown convincing results [30,37].

Based on the preclinical hypothesis as well as the Japanese data in several indications, the PHOENIX-01 trial was designed to evaluate carbon ion radiotherapy for patients with locally-advanced pancreatic cancer. Beam delivery will be performed using active rasterscanning, and the trial is designed as a dose-escalation trial.

In conclusion, this is the first trial to evaluate actively delivered carbon ion beams in patients with locally advanced pancreatic cancer within a dose-escalation strategy.

## Abbreviations

CT: Computer tomography; CTCAE: Common toxicity criteria for adverse events; CTV: Clinical target volume; DRR: Digitally reconstructed radiograph; EC: Ethics committee; GCP: Good clinical practice; GSI: Gesellschaft für Schwerionenforschung; GTV: Gross tumor volume; Gy: Gray; Gy E: Gray equivalent; LET: Linear energy transfer; MRI: Magnet resonance imaging; MTD: Maximum tolerated dose; OS: Overall survival; PFS: Progression-free survival; PR: Partial response; PTV: Planning target volume; QOL: Quality of life; RBE: Relative biological effectiveness; SD: Stable disease; RT: Radiation therapy; WHO: World Health Organisation.

## Competing interests

The authors declare they have no competing interests.

## Authors’ contributions

SEC, DH, MK, JW, MWB, DJ, OJ and JD wrote the study protocol and helped obtain the required votes of the relevant authorities. MK provided considerations on design and statistical issues and will follow the study and evaluation. SEC, DH, JW, MWB, DJ, RH and JD will provide patient care. OJ will perform treatment planning and plan data analysis. SEC, DH, JD and RH will implement the protocol and oversee collection of the data. All authors contributed to and approved the final manuscript version.

## Pre-publication history

The pre-publication history for this paper can be accessed here:

http://www.biomedcentral.com/1471-2407/13/419/prepub
